# Physiological, perceptual and neuromuscular responses of team sport athletes to short duration high intensity interval training using cycling

**DOI:** 10.1007/s00421-025-05803-4

**Published:** 2025-05-12

**Authors:** Craig Twist, Elliot Conboy, Max Davidson, Shane Price, Jamie Highton

**Affiliations:** 1 Research Institute of Sport and Exercise Sciences, Liverpool John Moores University, Liverpool, UK; 2 Division of Public Health, Sport and Wellbeing, University of Chester, Chester, UK

**Keywords:** Aerobic exercise, Physical conditioning, Metabolism, Muscle strength

## Abstract

**Purpose:**

To examine the acute physiological, perceptual and neuromuscular responses of team sport athletes to two volume-matched cycling high intensity interval training (HIIT) sessions with short work bouts (< 60 s).

**Methods:**

Using and randomised crossover design, 16 male team sport players completed 2 × 6 min (with 5 min between sets) repeated efforts of 15 s or 30 s exercising at 120% power at $\dot{\text{V}}$O_2 max_ (p$\dot{\text{V}}$O_2 max_) followed by matched-duration passive recovery on a cycle ergometer.

**Results:**

Absolute mean $\dot{\text{V}}$O_2_ (p = 0.0257) and relative mean $\dot{\text{V}}$O_2_ (p = 0.0275) were higher in 15 s than 30 s HIIT. Total time at > 90% $\dot{\text{V}}$O_2 max_ during the HIIT was higher for 15 s compared to 30 s HIIT (p = 0.0257). Heart rate remained the same between trials (p = 0.805) as did oxygen pulse (p = 0.1161). B[La] was lower in 15 s compared to 30 s HIIT (p = 0.0257). Differences in dRPE-L (p = 0.0495), dRPE-B (p = 0.0495) and dRPE-O (p = 0.1837) suggested lower perceived exertion in 15 s compared to 30 s HIIT. Maximal isometric knee extension force revealed a greater reduction after 30 s HIIT (p = 0.0495).

**Conclusion:**

Team sport athletes using short duration cycling-based HIIT should use 15 s work intervals to elicit greater time working near $\dot{\text{V}}$O_2 max_ at a lower perceived exertion and with smaller reductions in peak muscle force after exercise.

## Introduction

High intensity interval training (HIIT) offers a low volume, high intensity training approach comprising brief exercise bouts interspersed with periods of recovery. Prescription of HIIT involves manipulation of nine variables (e.g., exercise/recovery duration, intensity, modality, volume) to target specific central and peripheral adaptations (Buchheit and Laursen 2013a, b). Understanding how the manipulation of key HIIT variables influences the acute physiological response is important for those wishing to apply this training approach with athletes (MacInnis and Gibala 2017).

Short duration HIIT (i.e., < 60 s work/rest intervals) is a common training approach adopted by team sport athletes (Dupont et al. 2004; Buchheit et al. 2009). Closely replicating the demands associated with team sports (Dupont et al. 2004), short intervals enable the athlete to spend extended periods ~ 90%$\dot{\text{V}}$O_2 max_ (~ 50–60% total work time; Dupont et al. 2002) to drive central (i.e., oxygen delivery) and peripheral (i.e., oxygen extraction) adaptations associated with improved performance. Shorter intervals might also enable preservation of glycogen in favour of intramuscular triglycerides (Billat 2001) and a lower perceived effort compared to longer intervals (Valstad et al. 2018). While studies have reported lower oxygen uptake, heart rate, and blood lactate concentration for short (i.e., < 60 s) compared to long (i.e., > 60 s) HIIT matched for total work (Myrkos et al. 2022; Tschakert et al. 2015; Tucker et al. 2015), comparisons between HIIT in the same classification remain to be fully elucidated. Fernando Farias-Junior et al. (2019) compared 30/30 s and 60/60 s work/rest intervals whilst running on a treadmill, reporting a higher $\dot{\text{V}}$O_2_ in work bouts with longer intervals but a similar mean $\dot{\text{V}}$O_2_ for the overall exercise session. However, these data were collected in untrained participants and did not include shorter work bout durations consistent with those reported in team sports (i.e. ~ 15 s; Dupont et al. 2004; Buchheit et al. 2009). Further studies exploring how the manipulation of work bout duration can influence the acute training response are necessary to inform exercise prescription.

In a recent survey of team sport practitioners (Rogers et al. (2024), 99% of respondents reported the prevalence of non-specific forms of training, including cycle ergometry, as a supplementary training modality that accounted for ~ 20% of total training. Team sports practitioners adopting non-specific training with players will typically use HIIT comprising short duration (< 60 s) work intervals to target central and peripheral adaptations (Rogers et al. 2024). Cycle ergometry might also be adopted for those athletes who require more careful load management, e.g. during rehabilitation after injury or in the days after match play, or to provide alternative training stimuli (Mallol et al. 2020; Thom et al. 2020). However, the adoption of cycle ergometry with team sports athletes is interesting given their distinct response to HIIT using cycling and running (Twist et al. 2023) and that only two studies using this approach have reported improved intermittent running performance (Jones et al. 2015; Thom et al. 2020). Therefore, despite the prevalence of cycling-based HIIT using short duration intervals in team sport athletes (Rogers et al. 2024), further studies are required to elucidate the acute responses that will inform exercise prescription.

To inform the cycle ergometry training practices of team sport athletes, the purpose of this study was to conduct the first investigation into the acute physiological, perceptual and neuromuscular responses to volume-matched cycling HIIT sessions comparing 15 s and 30 s work intervals. We hypothesised longer duration intervals would elicit a higher $\dot{\text{V}}$O_2,_ heart rate, perceived exertion, blood lactate concentration and a greater reduction in muscle function after exercise compared to the shorter intervals.

## Methods

With institutional ethics approval (U23_SPS_3604), 16 male trained team sport players (age 20.9 ± 0.9 y, stature 180 ± 7 cm, body mass 75.7 ± 6.2 kg, $\dot{\text{V}}$O_2 max_ 50.6 ± 6.2 ml/kg/min, p$\dot{\text{V}}$O_2 max_ 301 ± 47 W) participated in this study after providing written informed consent. To ensure statistical power, an *a-priori* sample size calculation was performed based on observed differences in $\dot{\text{V}}$O_2_ (d_z_ = 3.08) and RPE (d_z_ = 1.33) with 30 s vs 60 s work intervals (Fernando Farias-Junior et al. 2019); this indicated that a minimum sample of 4–10 participants would be required. Our sample size was greater than the a priori calculation to account for participant attrition and to improve the generalizability of our findings. We also targeted several sports clubs during recruitment that resulted in a higher enrolment than anticipated. Participants played soccer and rugby to university or semi-professional standard once per week and completed team-sport training at least twice per week. All participants were familiar with using cycle ergometry, but none engaged frequently with cycling-based HIIT. Participants were asked to consume their normal pre-exercise diet and hydration before the first visit and asked to repeat this for all subsequent visits. Participants were also asked to avoid caffeine in the 2-h before each visit. Trials were performed at a similar time of day (± 2 h) with no vigorous physical activity in the 48 h before.

The study was conducted across two laboratories using the same procedures and the same equipment for both trials. Participants first attended the laboratory completing an incremental test to exhaustion on a cycle ergometer to establish power at maximal oxygen uptake (p$\dot{\text{V}}$O_2 max_) (Lode Medical Technology, Groningen, The Netherlands). The protocol started at 100 W and increased by 20 W/min until volitional exhaustion. Volitional exhaustion was defined as the point at which participants could no longer maintain a cycling cadence of 50 rev/min. Expired air was collected continuously throughout each trial using a pre-calibrated metabolic cart (Quark RMR, Cosmed, Cosmed.S.R.L., Italy or Metalyzer 3b, Cortex, Germany). Oxygen uptake ($\dot{\text{V}}$O_2_), was recorded breath-by-breath and later averaged over 30 s, with $\dot{\text{V}}$O_2 max_ accepted as the highest $\dot{\text{V}}$ O_2_ averaged over a 30 s epoch.

Participants completed two HIIT trials using either 15 s work/15 s rest or 30 s work/30 s rest intervals in a randomized crossover design, with 5–7 days between trials. Each HIIT session comprised 15 or 30 s at 120% p$\dot{\text{V}}$O_2 max_ (361 ± 56 W) followed by 15 or 30 s passive recovery, repeated for 6 min. Participants completed 2 sets with a 5 min recovery between each 6 min bout cycling at 40% p$\dot{\text{V}}$O_2 max_ (120 ± 19 W). Oxygen uptake (breath-by-breath) and heart rate were measured throughout, with values for mean $\dot{\text{V}}$O_2_ (absolute and relative maximum), time with oxygen > 90% $\dot{\text{V}}$O_2 max_ and oxygen pulse (Whipp et al. 1996) calculated. Blood lactate concentration (Biosen C-Line, EKF Diagnostic GmbH, Germany or Lactate Pro II, Arkray, Japan) was recorded immediately after with differential rating of perceived exertion (dRPE) for overall exertion (dRPE-O), breathlessness (dRPE-B) and leg-muscle exertion (dRPE-L) recorded 30 min after each HIIT trial using the Centimax scale (CR100; Borg and Borg 2002). Maximal voluntary isometric contraction of the knee extensors (MVC) in the dominant limb was measured immediately before and after each HIIT trial using a custom-built apparatus with the participant seated and the knee angle fixed at 90 degrees.

### Statistical analysis

To provide meaningful insight on the magnitude and probability of observed effects, all comparisons are reported as effect sizes (Cohen’s d; mean difference between trials/pooled standard deviation) and 95% confidence intervals (ES [95% CI]), with threshold values of 0.0–0.19, trivial; 0.2–0.59, small; 0.6–1.19, moderate; 1.2–1.9, large; ≧2.0, very large (Hopkins et al. 2009). These thresholds were used in the absence of accepted minimum thresholds for changes in the measurements of interest. Effects with confidence intervals that crossed a small positive or negative change were classified as unclear. This was accompanied by p-values based on appropriate null hypothesis tests, although any ES confidence interval that includes zero can be considered as p > 0.05. Data were checked for assumptions of normality using the Shapiro-Wilks test and were normally distributed (p > 0.05). Differences in physiological and perceptual responses were analyzed using separate paired-samples *t*-tests, with differences in RPE over time examined using a two-way repeated measures analysis of variance. To account for the increased risk of making a Type I error with multiple comparisons (family-wise error), the Benjamini–Hochberg method was used to adjust p-values using a false-discovery rate of 0.5%. Pearson-product moment correlations (r) and coefficients of variation (%CV) were also calculated to establish the influence of individual physical qualities and between-participant response to HIIT, respectively. All data were analysed using SPSS (version 27, Chicago, Illinois, USA).

## Results

**Fig. 1:**
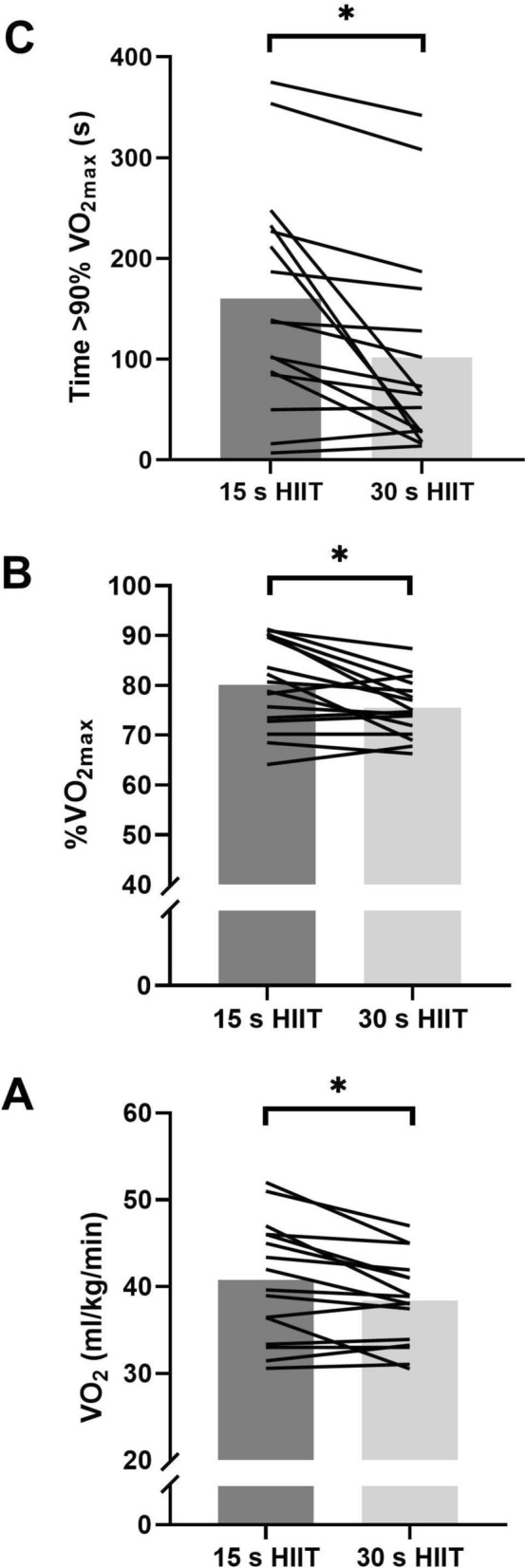
Differences in A) Mean $\dot{\text{V}}$O_2_, B) %$\dot{\text{V}}$O_2 max_ and C) Time > 90%$\dot{\text{V}}$O_2 max_ between 15 and 30 s cycle-based HIIT for team sport athletes (n = 16). *Indicates differences between trials with adjusted p values using a false-discovery rate of 0.5%

There were moderate differences in absolute (40.8 ± 6.8 cf. 38.4 ± 5.0 ml/kg/min; ES [95%CI] = 0.78 [0.20 to 1.33], p = 0.0257; Fig. 1A) and relative mean $\dot{\text{V}}$O_2_ (80.7 ± 9.8 cf. 76.1 ± 6.1%$\dot{\text{V}}$O_2 max_; ES [95%CI] = 0.74 [0.19 to 1.26], p = 0.0275; Fig. 1B), with 15 s HIIT higher than 30 s HIIT. Accordingly, total time > 90% $\dot{\text{V}}$O_2 max_ during the HIIT was moderately higher for 15 s compared to 30 s HIIT (176 ± 135 cf. 102 ± 106 s; ES [95%CI] = 0.83 [0.23 to 1.4], p = 0.0257; Fig. 1C). There were trivial differences in mean HR (159 ± 9 cf. 158 ± 14 bpm (79.7 cf. 79.4%_HRmax_); ES [95%CI] = 0.063 [−0.43 to 0.55], p = 0.805) between the 15 and 30 s HIIT trials. Likewise, differences in oxygen pulse were small between 15 and 30 s HIIT trials (19.5 ± 3.8 cf. 18.7 ± 4.2 ml/min; ES [95%CI] = 0.45 [− 0.08 to 0.95], p = 0.1161). RPE increased with time during both HIIT bouts (F = 47.2, p < 0.001) and was moderately higher in the 30 s compared to 15 s trials (15.9 ± 1.6 cf. 16.5 ± 1.7; ES [95%CI] = −0.71 [−1.25 to −0.15], p = 0.0286).

Correlations between $\dot{\text{V}}$O_2 max_ and total time > 90% $\dot{\text{V}}$O_2 max_ were r [95%CI] = −0.387 [−0.741 to 0.134] and r [95%CI] = − 0.369 [− 0.741 to 0.177] for 15 s and 30 s HIIT, respectively. The between-participant coefficient of variation (%CV) for total time > 90% $\dot{\text{V}}$O_2 max_ was 68% and 104% for 15 s and 30 s HIIT trials, respectively.

B[La] was moderately lower in 15 s compared to 30 s HIIT (ES [95%CI] = −0.88 [− 1.44 to − 0.28], p = 0.0257). Small to moderate differences in dRPE-L (ES [95%CI] = −0.58 [− 1.10 to − 0.04], p = 0.0495), dRPE-B (ES [95%CI] = −0.61 [− 1.13 to − 0.06], p = 0.0495) and dRPE-O (ES [95%CI] =  − 0.36 [− 0.86 to 0.15], p = 0.1837) indicated lower perceived effort in 15 s compared to 30 s HIIT. Data are shown in Table 1.

**Table 1:** Differences in blood lactate response (B[La]) and differential rating of perceived exertion (dRPE) for overall exertion (dRPE-O), breathlessness (dRPE-B) and leg-muscle exertion (dRPE-L) between 15 and 30 s cycle-based HIIT for team sport athletes (n = 16)

	15 s HIIT	30 s HIIT
B[La] (mmol/L)	8.4 ± 3.2*	10.6 ± 2.2
dRPE-L	65.2 ± 15.6*	73.3 ± 15.3
dRPE-B	59.7 ± 20.9*	70.1 ± 16.6
dRPE-O	65.3 ± 17.5	71.3 ± 15.5

*Indicates different to 30 s HIIT trial with adjusted p-values using a false-discovery rate of 0.5%

**Fig. 2:**
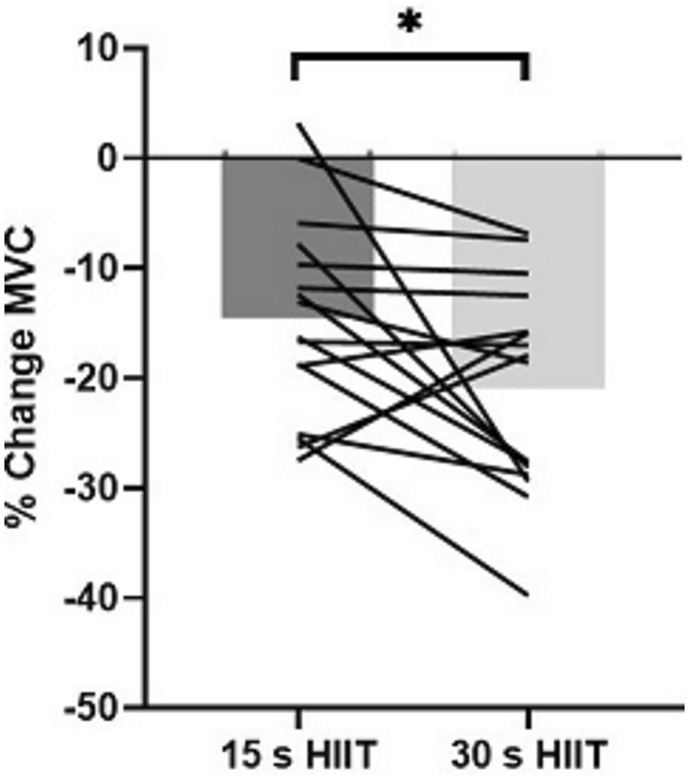
Changes in MVC (%) after 15 s and 30 s cycle-based HIIT for team sport athletes (n = 16). *Indicates differences between trials with adjusted p-value using a false-discovery rate of 0.5%

MVC was reduced after both 15 s (920 ± 376 to 730 ± 318 N) and 30 s (931 ± 377 to 718 ± 251 N) HIIT trials (p < 0.001), with a greater reduction after 30 s HIIT (∆%− 14.5 ± 9.2 cf. − 20.9 ± 9.6%; ES [95%CI] = − 0.58 [− 1.10 to − 0.04], p = 0.0495; Fig. 2).

## Discussion

To the authors’ knowledge, this is the first study to examine important acute physiological, perceptual and neuromuscular responses of team sport athletes to two short duration HIIT cycling training sessions. Despite high between-participant variability for both HIIT formats, shorter duration intervals comprising 15 s work/rest intervals resulted in a moderately higher mean $\dot{\text{V}}$O_2_ response and time spent > 90% $\dot{\text{V}}$O_2 max_ compared to 30 s intervals that were matched for overall work/rest time. Paradoxically, a moderately lower perceived exertion, lower blood lactate concentration, and smaller change in knee extensor MVC response was observed in the 15 s compared to 30 s intervals. Differences in mean heart rate and oxygen pulse were trivial between interval types.

Contrary to the hypothesis, our data suggest that cycling training at 120% p$\dot{\text{V}}$O_2 max_ using 15 s work/rest intervals elicits superior mean $\dot{\text{V}}$ O_2_ response and time > 90% $\dot{\text{V}}$ O_2 max_ in team sport athletes compared to intervals adopting a 30 s work/rest interval for the same total duration. A moderately higher mean $\dot{\text{V}}$O_2_ in the 15 s HIIT trial occurred despite trivial differences in heart rate and oxygen pulse data compared to the 30 s trial, which would suggest better peripheral extraction in the shorter trial. These findings reaffirm previous work using 30 s work/rest running intervals performed at 120% speed at $\dot{\text{V}}$O_2 max_ using a passive recovery (Fox et al. 1973) that resulted in $\dot{\text{V}}$O_2_ reaching only 70% of $\dot{\text{V}}$O_2 max_ during the work periods. Moreover, our data support Fernando Farias-Junior et al. (2019) who observed lower $\dot{\text{V}}$O_2_ during the recovery bout of longer running intervals that reduced the mean $\dot{\text{V}}$O_2_ for the session. Exercise performed in the lower portion of the extreme domain results in the highest mean $\dot{\text{V}}$O_2_ during the exercising period (~ 91% $\dot{\text{V}}$O_2 max_), with recovery $\dot{\text{V}}$O_2_ decreasing immediately after the termination of exercise (Ozkaya et al. 2023). Therefore, where a passive recovery is used between work intervals in the extreme domain, 15 s intervals are likely to allow less time for $\dot{\text{V}}$O_2_ to recover during the recovery interval facilitating a higher mean $\dot{\text{V}}$O_2_ for the session. The use of a passive recovery can also slow lactate removal between work intervals and might explain the higher blood lactate concentration for the 30 s compared to 15 s HIIT session (Billat 2001). Studies that explore 15 s and 30 s work bouts with active versus passive recovery are now warranted.

Where the purpose of training is to maintain or improve oxygen utilization and delivery, a key target for HIIT training sessions is to maintain more time with VO_2 _> 90% $\dot{\text{V}}$O_2 max_ to stimulate desired central and peripheral adaptations (Laursen and Jenkins 2002; Midgley and Naughton 2006). Target times of ~ 5–7 min have been proposed for team sport athletes using HIIT (Buchheit and Laursen 2013b; Dolci et al. 2020). The 15 s intervals elicited a moderately greater exercise time with the $\dot{\text{V}}$O_2_ response > 90% $\dot{\text{V}}$O_2 max_ than 30 s HIIT, representing 24 ± 15% and 14 ± 14% of total exercise time, respectively. These findings challenge the use of low volume (1–2 sets), short duration cycling HIIT for driving central and peripheral adaptations to improve $\dot{\text{V}}$O_2 max_ with team sport athletes.

Compared to short duration HIIT using running at similar intensities (Millet et al. 2003; Buchheit et al. 2009; Bok et al. 2023), we report a shorter time with $\dot{\text{V}}$O_2_ response > 90% $\dot{\text{V}}$O_2 max_. Some participants also recorded no or very limited time above the defined threshold during either HIIT session (Fig. 1C), with very large between-participant variability in time with oxygen demand > 90% $\dot{\text{V}}$O_2 max_. To maximise time > 90% $\dot{\text{V}}$O_2 max_ both exercise trials were performed at supramaximal exercise intensities above $\dot{\text{V}}$O_2 max_ based on the individual’s maximal aerobic power (Dupont et al. 2002; Millet et al. 2003). However, despite being a typical approach, prescribing exercise intensity based only on maximal aerobic power fails to account for an individual’s anaerobic power reserve (i.e., the difference between maximal aerobic power and maximal power output) that leads to large between participant variability in the response to supramaximal HIIT exercise (Bok et al. 2023). This variability is likely caused by differences in metabolic profiles between participants that direct the anaerobic and neuromuscular contribution to supramaximal HIIT (Sandford et al. 2021). While high variability in time > 90% $\dot{\text{V}}$ O_2 max_ seems normal, our values are higher than those reported by Bok et al. 2023; CV ~ 48%) during 15 s/15 s HIIT at 110% maximal aerobic speed and were larger for the 30/30 s trial. Such differences are possibly attributed to the mode of exercise, reaffirming previous studies that have reported differences in the physiological and neuromuscular response to short duration HIIT between cycling and running in team sport athletes (Twist et al. 2023). Compared to running, the oxygen response to cycling is slower (Hill et al. 2003) as is the time constant of the primary response when exercise intensity increases above $\dot{\text{V}}$O_2 max_ (Scheuermann and Barstow 2003). In addition, inadequate adjustment of the cardiovascular system and oxygen delivery (Scheuermann and Barstow 2003) and the insufficient intensity of the warm-up before exercise that would have impaired the oxygen response to the HIIT (Jones et al. 2003) could also explain both the high variability between individuals and the low time with oxygen demand > 90% $\dot{\text{V}}$ O_2 max_. Greater between participant variability for the longer trials supports our observations of a lower mean $\dot{\text{V}}$O_2_ for the session because 30 s intervals allow more time for $\dot{\text{V}}$ O_2_ to recover during the recovery interval. Future studies using exercise prescription that accounts for an individual’s anaerobic power reserve and exploring the oxygen kinetics to short duration HIIT during cycle ergometer training in team sport athletes are needed.

Overall perceived exertion was moderately lower for HIIT using 15 s rather than 30 s intervals, despite the same exercise intensity and same total work time. A lower perceived exertion could influence exercise tolerance (Marcora and Staiano 2010), particularly when performing repeated sets of high intensity intervals. This is particularly important for those team sport athletes that require an additional conditioning stimulus without high effort that might negatively influence task engagement, e.g., less fit athletes or those rehabilitating from injury. Taken together with the higher mean oxygen cost and more time near $\dot{\text{V}}$O_2 max_, shorter work/rest intervals might be useful when adopting cycling in team sport athletes to ensure the training is physiologically challenging and more tolerable for individuals.

A small increase in leg-exertion (dRPE-L) for the 30 s compared to 15 s HIIT trial accompanied a greater reduction in MVC for the longer trials, supporting the sensitivity of this perceived measure to differentiate specific inputs to exercise (Mclaren et al. 2016). While ventilation per se was not reported, a moderately higher perceived breathlessness (dRPE-B) for the 30 s compared to 15 s trial likely reflects the greater ventilation to address the higher blood lactate concentration and manage the acid–base balance during longer HIIT durations. Differences in overall dRPE (dRPE-O) were trivial between the 15 s and 30 s HIIT for which the precision of the estimate included zero. Our findings support the use of differential RPE to provide a sensitive measure of internal load that differentiates between the specific central and peripheral inputs during HIIT in team sport athletes (Mclaren et al. 2016).

Understanding the neuromuscular response to HIIT is important since it can influence exercise performance and determine the potential interference on subsequent training and increased injury risk (Buchheit & Laursen 2013b). Reductions in knee extensor peak force occurred after both 15 s and 30 s HIIT trials. Together with the data described above, these findings support the notion that short duration HIIT challenges both the neuromuscular as well as that of the cardio-respiratory systems (Buchheit & Laursen 2013b). A greater reduction in peak torque occurred after 30 s HIIT, which was also accompanied by a higher perceived effort during the task, a higher differential rating of perceived exertion for the limbs and a higher blood lactate concentration after exercise. A greater reliance on non-oxidative metabolism during 30 s efforts, evidenced indirectly by a higher blood lactate concentration, would suggest the potential for a greater reduction in muscle pH after HIIT (Glaister 2005). The higher accumulation of metabolites and substrate depletion after 30 s HIIT are likely to have contributed to fatigue and the observed reduction in maximal voluntary contraction (Allen et al. 2008). Reduced central nervous system activation is also a likely candidate to explain a loss in peak force (Taylor and Gandevia 2008) but is difficult to confirm without further investigation. Future studies to uncouple the central and peripheral mechanisms between different duration HIIT protocols are needed.

## Limitations

While trained, our participants were not performing at the highest standard. Further study is needed with elite team sport athletes to understand their response to cycle ergometer training. We also acknowledge that our study reports on the acute responses which might not predict an individual’s chronic adaptation to short duration HIIT. As already alluded to, the use of maximal aerobic power to establish exercise intensity meant our training prescription did not account for the individual’s anaerobic power reserve. This means those with a lower peak power would have completed HIIT at a higher proportion of their anaerobic power reserve, which might have influenced some physiological and perceived responses (Bok et al. 2023). Finally, more invasive approaches are required to fully elucidate the mechanisms responsible for the observed response to cycle ergometer training in team sport athletes.

## Conclusions

We offer insight to the physiological, perceptual and neuromuscular response of team sport athletes to short duration HIIT using cycle ergometry. These findings are important given the prevalence of this training approach in team sports. When using short duration cycling HIIT with team sport athletes, a higher mean $\dot{\text{V}}$O_2_ for a lower perceived exertion, lower blood lactate concentration and lower neuromuscular load were observed for 15 s compared to 30 s work/rest intervals. Using 15 s work/rest intervals might enable athletes to maximise the mean $\dot{\text{V}}$O_2_, time near mode-specific maximum aerobic capacity alongside a lower perceived and neuromuscular load during short duration cycling HIIT. When using short duration cycling HIIT, practitioners should be mindful of losses in knee extensor muscle strength immediately after HIIT and that these reductions might be greater after longer intervals. Those using low volume, short duration cycling HIIT with team sports athletes should be aware of the high between-athlete variability in the time with oxygen demand > 90% $\dot{\text{V}}$O_2 max_. This will be influenced by differences in an individual’s anaerobic and neuromuscular contribution to supramaximal HIIT and has implications for how exercise intensity for this training modality is prescribed. Future research should explore the effect of short duration cycle ergometry training approaches of different durations and how these might be manipulated to target specific chronic physiological and performance adaptations in team sport athletes. Future work should also try to elucidate the underpinning mechanisms that explain individual responses to short duration HIIT to optimise their use in exercise prescription.

## Data Availability

The data from the current study are available from the corresponding author on reasonable request.

## Abbreviations

B[La]: Blood lactate concentration
dRPE: Differential rating of perceived exertion
ES: Effect size
HIIT: High intensity interval training
HR: Heart rate
MVC: Maximal voluntary isometric contraction
p $\dot{\text{V}}$ O_2 max_: Power at maximum oxygen uptake
RPE: Rating of perceived exertion
$\dot{\text{V}}$ O_2 max_: Maximum oxygen uptake
$\dot{\text{V}}$ O_2_: Volume of oxygen
